# Supplementary material for: Possible Epigenetic Origin of a Recurrent Gynandromorph Pattern in Megachile Wild Bees
Source: Insects. 2021 May 12;12(5):437. doi: 10.3390/insects12050437 (PMC8151954; doi:10.3390/insects12050437)
Supplement: Supplementary file 1 [file insects-12-00437-s001.zip › insects-1195579-supplementary.pdf]

Table S1. List of *Megachile* transverse gynandromorphs discussed in the paper. References as in the main text.

Species	N. Sp.	Locality	Reference
M. (Chelostomoides) angelarum Cockerell, 1902	2	Piney Valley, California, USA<br>Twain Harte, California, USA	[15]
M. (Ptilosaurus) bertonii Schrottky, 1908	2	Chapada, Brazil	[14]
M. (Pseudocentron) curvipes Smith, 1853	1	S. Geronimo, Guatemala	[15]
M. (Eutricharaea) deceptoria Pérez, 1890	1	Tarhankut Peninsula, Ukraina	[9]
M. (Eutricharaea) detersa Cockerell, 1911	15	Not reported	[22]
M. (Eutricharaea) gathela Cameron, 1908	1	Kangra, India	[28]
M. (Xanthosaurus) gemula Cresson, 1878	1	Bryson City, North Carolina, USA	[14]
M. (Leptorachis) intergradus Mitchell, 1929	2	Pedra Brancha, Brazil<br>Chapada, Brazil	[14]
M. (Eutricharaea) cf. *leachella* Curtis, *1828*	1	Andritsaina, Greece	[68]
M. (Xanthosaurus) maritima (Kirby, 1802)	1	Berskow, Germany	[26]
M. (Austromegachile) montezuma Cresson, 1878	1	São Carlos, Brazil	[27]
M. (Litomegachile) onobrychidis Cockerell, 1914	1	Lompoc, California, USA	[14]
M. (Chelostomoides) otomita Cresson, 1878	1	Pujiltic, Chiapas, Mexico	[8]
M. (Argyropyle) parallela Smith, 1853	1	Lane Co, Kansas, USA	[14]
M. (Argyropyle) parallela Smith, 1853	1	Twain Harte, California, USA	[15]
M. (Xanthosaurus) perihirta Cockerell, 1898	1	Mt. Hood, Oregon, USA	[14]
M. (Eutricharaea) picicornis Morawitz, 1853	6	Karadag, Crimea, Ukraine<br>Opuk, Crimea, Ukraine	[9]
M. (Eutricharaea) pilidens Alfken, 1924	6	Berici Hills, Italy	Here
M. (Eutricharaea) pilidens Alfken, 1924	1	Platania Volos, Greece	[23]
M. (Pseudocentron) rubricata Smith 1853	5	Minas Gerais, Santana do Riacho, Brazil	[29]
M. (Chrysosaurus) tapytensis Mitchell, 1929	1	Tapyta, Paraguay	[14]
M. (Neochelynia) uniformis Mitchell, 1929	1	Chapada, Brazil	[14]
M. (Xanthosaurus) vidua Smith, 1853	2	Bilby, Alberta, Canada<br>Lethbridge, Alberta, Canada	[14]
M. (Xanthosaurus) vidua Smith, 1853	1	Nordegg, Alberta, Canada	[15]
Megachile sp.	1	Chapada, Brazil	[14]
